# ‘If You Cut Me, I’d Probably Bleed Coca‐Cola’: A Qualitative Exploration of Barriers and Facilitators to Type 2 Diabetes Management Among Chinese Adults in the UK

**DOI:** 10.1155/jdr/8119786

**Published:** 2026-04-03

**Authors:** Tarnjit Sehmbi, Ran Cao, Raju Sapkota, Shahina Pardhan

**Affiliations:** ^1^ Vision and Eye Research Institute, School of Medicine, Anglia Ruskin University, Cambridge, UK, anglia.ac.uk; ^2^ Faculty of Business and Law, Anglia Ruskin University, Cambridge, UK, anglia.ac.uk

**Keywords:** barriers, Chinese, collectivist, diabetes, facilitators, lifestyle, type 2 diabetes, UK

## Abstract

**Objectives:**

Chinese populations are at an elevated risk of type 2 diabetes mellitus (T2DM) and related complications, yet limited research has explored diabetes self‐management in the UK Chinese community. This study aimed to explore barriers and facilitators to T2DM self‐management in the UK Chinese community.

**Design:**

This study adopted a qualitative design. Three focus group discussions (FGDs) were conducted on Zoom with 22 Chinese participants with self‐reported type 2 diabetes living in the UK. The participants ranged from 24 years to 85 years (mean age = 66 years, SD = 17.1). A purposive sample was recruited through study adverts in Chinese community centres and via snowball sampling. Data were analysed using Braun and Clarke’s thematic analysis (TA).

**Results:**

This paper discusses four themes in detail. (1) Key barriers to healthy eating were time constraints and convenience, cultural expectations and lack of nutritional knowledge. (2) Facilitators for healthy eating included having a support network, health education and modifying eating habits. (3) Barriers to physical activity included low importance of exercise, work and family commitments and environmental constraints. (4) Facilitators for physical activity were community‐based exercise, culturally appropriate exercise such as Tai Chi, and benefits to mental health. It was found that dietary changes were perceived as important in diabetes management; however, physical activity was seen as less important when considering diabetes management.

**Conclusion:**

Diabetes self‐management among UK Chinese adults is shaped by collectivist family structures, culturally embedded food practices, and barriers within healthcare provision. Interventions should move beyond individualised behaviour change models and incorporate family‐inclusive education, bilingual resources, and community‐embedded physical activity programmes. Culturally appropriate diabetes services may enhance engagement and support self‐management within this population.

## 1. Introduction

Type 2 diabetes mellitus (T2DM) is a chronic metabolic disorder characterised by hyperglycaemia resulting from insulin resistance and/or deficiency [1]. It is associated with an increased risk of serious health complications, including cardiovascular disease, kidney disease and vision loss [2, 3]. The condition is highly prevalent, accounting for over 90% of all diabetes cases [4, 5]. The International Diabetes Federation estimated the total number of people with diabetes at 463 million cases of diabetes worldwide in 2019, with the number projected to rise to 643 million by 2030 and 783 million by 2045 [6].

Ethnic minority populations have a higher risk of developing type 2 diabetes and its complications. In particular, individuals of South Asian, Chinese and African or African‐Caribbean descent are two to four times more likely to develop diabetes compared to White populations [7, 8]. In the UK, Chinese people represent ~0.7% of the population in England and Wales, with higher concentrations in cities such as Cambridge and London [9]. Despite this, little is known about the lived experiences of diabetes management in this population. While some epidemiological data have explored prevalence and risk factors for T2DM in Chinese communities globally, including in Singapore, Hong Kong and mainland China [10, 11], there is a notable lack of qualitative research exploring the experiences of Chinese people living with type 2 diabetes in the UK.

Environmental and lifestyle factors, including the adoption of westernised diets rich in animal fats and processed carbohydrates, have been shown to contribute to the rising prevalence of diabetes in Chinese and other ethnic minority groups [12]. Physical inactivity is another important factor, with Chinese populations both in China and abroad showing a decline in fitness and an increase in overweight/obesity despite modest improvements in leisure‐time activity [13, 14].

Sociocultural factors also shape how diabetes is experienced and managed. These include cultural dietary preferences, health beliefs, intergenerational communication and the use of traditional medicine [15–17]. However, most of the existing literature focuses on Chinese populations outside the UK, and no UK‐based qualitative studies to date have examined how Chinese people with T2DM understand and manage their condition within the UK healthcare context.

Uncontrolled diabetes can lead to a range of complications, including retinopathy, neuropathy, nephropathy, cardiovascular disease and lower‐limb problems [18–20]. Large‐scale studies conducted in China report that more than half of individuals with T2DM experience at least one chronic complication [21, 22].

Given the rising prevalence of type 2 diabetes among Chinese people and the risk of serious health outcomes if not well managed, it is crucial to understand how cultural, behavioural and healthcare system‐related factors influence disease self‐management. Yet, within the UK, there is a significant gap in the literature exploring the knowledge, awareness and lifestyle practices of Chinese people living with T2DM.

This study aims to explore the barriers and facilitators that influence diabetes self‐management among Chinese individuals diagnosed with type 2 diabetes living in the UK. It focuses on modifiable lifestyle behaviours: diet and physical activity. By addressing this gap, the study seeks to provide culturally grounded insights to help healthcare professionals tailor diabetes care and education in ways that are more inclusive and responsive to the needs of the Chinese community in the UK. Ultimately, the findings may inform the development of culturally appropriate prevention and intervention strategies to reduce the risk of diabetes‐related complications in this population.

## 2. Materials and Methods

### 2.1. Ethical Approval

Ethical approval was obtained from the Faculty of Medical Science Research Ethics Panel, Anglia Ruskin University prior to data collection (Approval Number ETH2223‐9252).

### 2.2. Design

This study adopted a qualitative design using focus group discussions (FGDs) to explore barriers and facilitators of diabetes self‐management in the Chinese community. FGDs were conducted instead of individual interviews due to the collectivist nature of Chinese culture, where shared discussion often encourages active participation, promotes community‐oriented dialogue and yields richer data [23, 24].

Three FGDs were conducted, comprising 4–11 participants each. Group composition included literate Chinese adults and those requiring language support from a community champion. All FGDs were conducted online via Zoom [25] between October and December 2023. Holding sessions at different times of the day accommodated participants’ varying schedules.

Information power conceptualises sample adequacy as dependent on study aim, participant specificity, dialogue quality and analytic depth [26]. Recruitment was concluded once the dataset had sufficient depth and richness to address the study aim, hence information power had been reached.

### 2.3. Participants: Sampling and Recruitment

Purposive sampling recruited a total of 22 participants (6 males and 16 females) to the study. Ages ranged from 24 to 85 years (mean age = 66 years, SD = 17.1) (See Table 1). All participants identified as Chinese and had lived in the UK for at least 10 years. The median duration of type 2 diabetes diagnosis was 8 years (range: 2–16 years). Participants were geographically distributed across Cambridge and Liverpool.

**Table 1:** Table of participant demographic variables.

Participant number	Gender	Age
FG1		
Participant 1	Female	24
Participant 2	Male	42
Participant 3	Male	54
Participant 4	Female	61
Participant 5	Female	37
FG2		
Participant 1	Female	61
Participant 2	Female	58
Participant 3	Female	58
Participant 4	Female	53
Participant 5	Male	53
Participant 6	Female	76
FG3		
Participant 1	Female	76
Participant 2	Male	81
Participant 3	Female	84
Participant 4	Female	76
Participant 5	Female	77
Participant 6	Female	78
Participant 7	Female	82
Participant 8	Female	72
Participant 9	Male	85
Participant 10	Male	84
Participant 11	Female	80

Participants were recruited using a pragmatic, multifaceted approach due to the known challenges of recruiting from ethnic minority populations, including mistrust and fear of exploitation [27]. Recruitment methods included study advertisements placed in Chinese community centres, word of mouth, social media groups and outreach through existing community networks. Previous participants from earlier studies who had consented to future contact were also invited. While the inclusion of these individuals may introduce a degree of selection bias, efforts were made to ensure diverse representation through broader community outreach. These returning participants were not overrepresented, and their perspectives were considered alongside those of newly recruited individuals. While email was the primary form of communication, telephone support was available for participants with limited digital access. Once consent had been obtained, participants were sent an invitation to an online FGD via email.

Participant information sheets and consent forms were provided in both English and Simplified Chinese. Where needed, verbal explanation in Cantonese or Mandarin was also offered by the researcher (Ran Cao). Participants were given time to ask questions and informed that participation was voluntary and could be withdrawn at any time without consequence.

The inclusion criteria required participants to be aged 18 years or older, of Chinese ethnic origin, currently living in the UK (for more than 1 year) and self‐reporting a diagnosis of type 2 diabetes. Exclusion criteria included not identifying as Chinese, having a diagnosis other than type 2 diabetes (e.g., type 1 or gestational diabetes) or not residing in the UK.

### 2.4. Data Collection

A semi‐structured topic guide was used to lead the FGDs, developed to explore key themes, including knowledge and perceptions of diabetes, cultural influences, healthcare access, dietary practices and physical activity. The same guide was used across all three groups.

Each group was facilitated by two researchers, one holding insider status as a Chinese female and one outsider, promoting cultural competence and reflexivity. All facilitators were experienced in qualitative methods. Discussions were audio‐recorded using Zoom’s built‐in feature and lasted ~60–90 min.

Focus groups were conducted in English, Mandarin or Cantonese, depending on participants’ preferences. Researcher Ran Cao and a translator in FG3, fluent in these languages, translated and transcribed the non‐English discussions into English. English‐speaking groups were transcribed verbatim by researcher Tarnjit Sehmbi. Transcription was done manually to preserve nuance and enhance familiarity with the data. This is also advantageous for data analysis [28]. NVivo V.12 was used to manage data coding and analysis.

### 2.5. Data Analysis

Data were analysed using thematic analysis (TA) [29], following the six‐phase process outlined by Braun and Clarke (2006): (1) Familiarisation with the data, (2) generating initial codes, (3) searching for themes, (4) reviewing themes, (5) defining and naming themes and (6) producing the final report. An inductive form of TA, which assumes no prior themes or framework of the data, was applied to allow emergence of ‘data‐driven’ themes [30]. TA was deemed as an appropriate method of data analysis due to its flexible nature [30]. Codes were identified at the semantic level, mirroring participants’ language and experiences [30]. A constant comparative method was employed, allowing iterative refinement of codes and themes during data collection and analysis [30, 31].

### 2.6. Trustworthiness and Rigour

To enhance the trustworthiness of the analysis, two researchers (Tarnjit Sehmbi and Ran Cao), both experienced in qualitative methodologies, independently coded 10% of the transcripts. Peer examination of data has been shown to strengthen credibility and dependability in qualitative research [32]. Any discrepancies in coding were discussed and resolved through consensus. A constant comparative approach was employed, with data analysis conducted concurrently with data collection. This iterative process enabled researchers to reflect on emerging insights and refine subsequent data collection and interpretation [22, 32].

### 2.7. Reflexivity

Reflexivity, the process of critically reflecting on the researchers’ positionalities and potential influence on the research process and interpretation of findings, was integral to this study [33, 34]. One researcher (Ran Cao) held an ‘insider’ status, sharing cultural and linguistic backgrounds with the participants, which provided nuanced insight into cultural norms and practices relevant to the study. While this facilitated rapport and contextual understanding, it also posed the potential for bias in interpretation. To mitigate this, a second researcher (Tarnjit Sehmbi), who held an ‘outsider’ status, independently coded a transcript to provide an alternative perspective [35]. Reflexive practices were embedded throughout the study: field notes were recorded after each focus group session, and regular debriefing discussions were held among the research team. These strategies promoted critical reflection and transparency in the analytic process [36], thereby enhancing the trustworthiness and credibility of the findings [37].

## 3. Results

Analysis of the focus groups using Braun and Clarke’s TA led to the identification of four themes (see Table 2). These themes are discussed below, along with illustrative quotes.

**Table 2:** Table of key themes and sub‐themes.

Barriers to healthy eating	Facilitators for healthy eating
1. Time constraints and convenience 2. Cultural expectations 3. Lack of nutritional knowledge	1. Having a support network 2. Health education 3. Modifying eating habits
Barriers to physical activity	Facilitators for physical activity
1. Low importance of exercise 2. Work and family commitments 3. Environmental constraints	1. Community‐based exercise 2. Culturally appropriate exercise 3. Benefits to mental health

### 3.1. Theme 1: Barriers for Healthy Eating

#### 3.1.1. Subtheme 1: Time Constraints and Convenience

Participants recognised that their current busy daily routines and work commitments often meant that they were neglecting their dietary health and acknowledged that this led to convenience eating rather than having balanced meals:

*I work in my restaurant so I eat when I get a chance* [*…*] *I don’t have a set lunch as such* [*…*] *I just don’t have the time* [*P2*, *FG1*]

Participants also described skipping breakfast due to a lack of time in the mornings and replacing this with a drink:

*I skip breakfast*, *just take a drink and then go straight to work* [*P1; FG1*]

Work commitments also meant participants were not eating regularly throughout the day:

*I don’t eat regular because I work quite sort of demanding work* [*…*] *probably not have regular eating* [*P1; FG1*]

Participant 5 reflected on their time in Hong Kong, and compared the availability of foods in the UK and Hong Kong, which influences convenience eating:

*In the UK there is no many options for me because I used to eat Keto* [*…*] *in the UK they eat many bread sandwich because there so many* [*P5; FG2*].

#### 3.1.2. Subtheme 2: Cultural Expectations

Several cultural factors which influence dietary behaviours were discussed by participants. Participants expressed that food played an important role in Chinese culture. Food was linked to social status when it came to weddings, and families were often expected to put on lavish spreads of food:

*To be seen as wealthy and successful we use food as an indicator of status at weddings and events and other festivals so food is very important to us* [*P5*, *FG2*]

Additionally, participants explained that although efforts were commonly made to improve their diet following a diagnosis of diabetes, Chinese festivals made this difficult. Chinese festivals were perceived as a time to celebrate and indulge in sweet foods, and some participants were happy to partake in eating large amounts of sweet foods only at Chinese festivals:

*They* [*festivals*] *come around once in a while* [*…*] *Festival is not usual*, *so we eat freely* [*P5*, *FG3*].

The social pressure to eat food when attending events was also discussed, as eating food that has been prepared for a festival can be seen as a sign of respect:

*I got married last year and my parents were actually very worried about the Chinese banquet ‘*cos *my dad was like I actually can’t really eat most of this stuff* [*…*] *But he also didn’t want to seem disrespectful by not enjoying the Chinese wedding feast* [*P5*, *FG1*, *referencing her father*].

#### 3.1.3. Subtheme 3: Lack of Nutritional Knowledge

When it came to foods that make up a balanced diet, a lack of nutritional knowledge was apparent within the participant group. Participants experienced challenges accessing diabetes‐specific information due to challenges such as language barriers:

*We are ethnic Chinese*, *we don’t understand English*, *so there is no way for us to know the information* [*p5; FG3*]

Additionally, participants reported that when they were shopping for food, they were unaware of the foods they should pick, as there was not specific information for diabetics on food packaging:

*I know there is a traffic system for food shown level*, *carbohydrate*, *sugar*, *salt all that stuff* [*…*] *But it’s not enough on diabetic food* [*P6*, *FG2*]

The lack of culturally appropriate dietary advice given to participants in GP appointments was commonly discussed, with participants reporting GPs and dieticians not being aware of the culturally appropriate foods for the Chinese community when giving dietary advice:

*When the dietitian comes in to try to help you It’s quite difficult sometimes since they don’t have the knowledge of what type of food we eat*. [*…*] *when we ask*, *oh*, *if is it okay for us to eat this food like it’s not too high in sugar or something like that they sometimes don’t give us the answer because they don’t know* [*P3*, *FG1*].

### 3.2. Theme 2: Facilitators for Healthy Eating

#### 3.2.1. Subtheme 1: Having a Support Network

Having a support network at home and in the workplace was seen as a facilitator for healthy dietary behaviours. A participant explained that since both her and her mum had been diagnosed with diabetes, they worked together to improve their knowledge about good diet and had in turn educated other family members on the dangers of diabetes and the importance of healthy changes to diet. This way, the management of diabetes becomes a shared and supportive task within a family:

*My parents are both type 2 diabetics* [*…*] *I lived with her sort of making changes to her diet*, *which kind of fell on myself and my dad* [*…*] *we all ate healthier which made it better* [*P4*, *FG2*]

Participants discussed how colleagues made provisions for their diabetes at social events and during festivals such as Christmas, promoting a supportive environment for their diabetes management:

*I feel warm just because they* [*colleagues*] *will buy some biscuit with no sugar in them me*, *so that I can eat with my diabetes* [*P5*, *FG2*]

Although some participants discussed the pressure to eat sugary foods at Chinese festivals and occasions, they also explained having other people at events who supported them made making healthy choices easier:

*I tell people about my diabetes at events because then they aren’t as pushy when I refuse certain foods I think it’s good when people understand that I’m making the choice for my health* [*P3*, *FG1*].

#### 3.2.2. Subtheme 2: Health Education

Lack of knowledge around diet encouraged participants to reflect on where they had got their health education from. Most commonly, this was shared amongst families or resources found online, and less often given from health professionals:

*Sometimes I cannot understand what the GP is telling me. I have WhatsApp with my friends and family and we talk about what to eat* [*P6*, *FG3*].

Some participants reported using apps such as WeChat, used almost exclusively by the Chinese community as a social communication platform.

*I learned from the information provided in the WeChat that the eating order is important* [*P9*, *FG3*]

Some community centres in the UK also hosted events on different topics of health to support the Chinese community:

*We have a Chinese community*, *which usually have some events or activities to help us to learn diabetes*, *and had a lecture about dementia* [*P4*, *FG3*].

#### 3.2.3. Subtheme 3: Modifying Eating Habits

Participants explained that they had either made a change or were intending to make a change to their eating habits to help manage their diabetes. It was acknowledged that dietary changes were required in order to reduce the impact of diabetes:

*I do need to reduce my sugar* [*…*] *If you cut me*, *I’d probably bleed Coca-Cola* [*P2*, *FG1*]

Participants discussed making small changes and replacing foods with healthier alternatives to manage their diabetes rather than making extreme changes to their diet such as cutting out whole food groups:

*We’ve been changing the rice from pure white rice to multi-grain rice to reduce the blood sugar levels* [*…*] *I don’t cut out all carbs* [*P3*, *FG1*]

Some participants had made changes to their eating habits for example eating more vegetables, not eating carbohydrates at night, controlling portion sizes, altering eating windows, as well as limiting staple foods such as rice:

*I will have white rice at lunch If I’m having rice but it will just tend to be a slightly smaller portion than maybe I was used to in the past* [*P5*, *FG1*]

Staple foods such as rice were kept in diets, as carbohydrates were perceived to keep participants full for longer, therefore, modifications were made to routines or other food groups such as proteins:

*I have a large portion of protein*, *and vegetables and little rice but I don’t eat rice at night anymore* [*…*] *I still like to eat little rice because it keep me full* [*P3*, *FG2*]

While most participants emphasised dietary modifications, such as reducing sugar and controlling portion sizes, as central to managing their diabetes, a deviant case emerged. One participant acknowledged the health risks of smoking but felt it was acceptable to continue because they had already made dietary changes to manage their condition:

*‘I know smoking is bad for diabetes, but I get used to smoking, can’t get rid of smoking […] I don’t want to get rid of smoking because I don’t eat too much’* [*P2*, *FG3*].

This account highlights variation in risk management strategies within the community and suggests that some individuals may prioritise dietary control over other lifestyle behaviours, such as smoking cessation, when managing diabetes.

### 3.3. Theme 3: Barriers for Physical Activity

#### 3.3.1. Subtheme 1: Low Importance of Exercise

In contrast to dietary changes, where participants had expressed a desire to eat healthier foods in order to manage their diabetes, participants were less willing to make changes in their levels of physical activity:

*I could sacrifice changing my diet and being healthy that way*, *but I just can never get my head round the exercise like I just didn’t want to do it* [*P5*, *FG2*]

This was generally due to less importance being placed on exercise, less recognition of the lack of exercise being a contributing factor towards diabetes onset; and the views participants held about exercise, due to a number of factors:

1. Prioritising work:

*Working is quite demanding so doing exercise* [*…*] *I do not think it is that important* [*P1*, *FG1*]

2. Exercise was perceived as a waste of time, as it could be used for more productive tasks such as preparing dinner and spending time with the family:

A*fter work is my family time so I wouldn’t use that to go to the gym* [*P2*, *FG1*].

Participants reflected that the elderly people in their family would find it difficult to understand why someone would ‘walk for fun’.

*My father had diabetes we tried to encourage him to go out for a walk* [*…*] *I don’t know whether it’s a generation thing*, *but he didn’t understand the need* [*…*] *if I was to just suggest to go to a park and go for a walk he would say why would I want to do that for* [*P2*, *FG1*].

#### 3.3.2. Subtheme 2: Work and Family Commitments

Work was not only seen as a time constraint for physical activity but also as a form of physical activity in itself. Several participants, especially those in physically demanding jobs such as catering, considered their occupational activity sufficient:

*‘I don’t personally do exercise, per se, as in going to the gym, but because of my long working hours, I’m doing 15 to 25 thousand steps walking around the kitchen and doing different things*’ [*P2*, *FG1*]

Some participants expressed that they had other family commitments which were more important than getting exercise, which they valued:

*I pick the grandchildren from school and drop them off classes after school so have no time for this* [*exercise*] [*P7*, *FG3*].

#### 3.3.3. Subtheme 3: Environmental Constraints

Additionally, participants discussed environmental constraints for their preferred physical activities. Examples of this are not being able to partake in activities such as table tennis and badminton due to the frequent windy weather in the UK; therefore, participants’ options of physical activity were often limited.

*In Hong Kong China I play badminton but here the weather too windy so I can not play* [*P5*, *FG2*]

Often, participants preferred activities such as Tai Chi and square dancing; however, the provision of these classes is limited:

*Before COVID-19 we had tai chi classes on a Tuesday but now they stopped not enough people came* [*P1*, *FG1*]

Geographical variations were also discussed. A participant compared the opportunity for physical activity in the UK and Hong Kong, China, explaining that in Hong Kong there were many opportunities to go for outdoor walks or engage in outdoor exercise; however, due to the weather in the UK, and the normalisation of driving, exercising outdoors was less appealing:

*I do less exercise in UK compared with when I in Hong Kong I think the main reason is because in UK we often drive point to point* [*…*] *but in Hong Kong I’m most likely I will walk*, [*…*] *in UK*, *I think because of weather it is much cooler than Hong Kong*, *so that make me lazy and also the weather in UK* [*…*] *it’s often raining So this discourage me to go out and do exercise* [*P5*, *FG2*].

### 3.4. Theme 4: Facilitators for Physical Activity

#### 3.4.1. Subtheme 1: Community‐Based Exercise

Participants preferred community‐based exercise where they could exercise with friends and family. Some forms of exercise were popular amongst participants, such as attending exercise groups held in community centres:

*I sometimes go to the elderly centre*, *play Tai Chi*, *dance*, *sing karaoke*, *and play Mahjong* [*P1*, *FG3*].

The sense of togetherness and support was a driving factor for physical activity among participants. It was a time to socialise and engage in something fun with friends and family:

*I like to go see my friend when I am doing Tai Chi because we have fun* [*P3*, *FG3*, *age 84*]

Additionally, participants expressed feeling motivated to exercise when it involved friends or family, suggesting support encouraged engagement in physical activity:

*if my partner or my wife would like to do exercise with me*, *that I think it will encourage me to do more exercise* [*P5*, *FG1*]

Participants also expressed that they would be more inclined to attend a group‐based activity due to the commitment made to the team, and not wanting to let the team down:

*If you’ve got a group of people doing sports with you’re more inclined to go* [*…*] *Say*, *if you playing football. you will go because there’s a group there waiting for you*. [*…*] *It’s not just for yourself*, *you’ve got the team to think about* [*P1*, *FG2*]

Overall, participants value group‐based activities and suggested that the government should arrange regular group activities for minority groups, along with a support group for diabetics to discuss diabetes management:

*A class for diabetics once a week*, *once a week at local gym*. [*…*] *they join*, *go together at a fixed time*, *do a session. They could talk about their diabetes and how to handle it and at same time do a bit of exercise for everyone. maybe that will form a group gathering while they’re able them to participate*. [*P6*, *FG2*].

#### 3.4.2. Subtheme 2: Culturally Appropriate Exercise

Participants explained that growing up, they had partaken in many group activities as encouraged by their parents, such as ballet classes. However, as they had grown up, they had gravitated towards sports such as badminton and tennis, which were seen as culturally appropriate sports:

*The younger generation they go to do dance when young* [*…*] *and I think table tennis and badminton is a very popular kinds of exercise for Chinese* [*P1*, *FG1*]

Activities such as walking at a slow pace and Tai Chi were enjoyed as a group within community centres. This encouraged a sense of community as well as preserving cultural heritage:

*We have regular Tai Chi in the Chinese community centre it is good for us and part of my culture* [*P4*, *FG3*].

*Walking is very Chinese way of exercise* [*…*] *maybe doing that as more of like a day out trip and not big exercise* [*P1*, *FG1*]

Furthermore, participants were willing to travel to engage in physical activity that was culturally appropriate and where they could meet and socialise with other Chinese people:

*I travel across* [*city name*] *to play table tennis at a Chinese community* [*…*] *it’s something that gets me going*, *and I enjoy it*. [*P2*, *FG2*].

#### 3.4.3. Subtheme 3: Benefits to Mental Health

Although exercise was commonly perceived as less important than other commitments such as work and spending time with family, several participants discussed the mental health benefits of walking. This was especially relevant in the context of the COVID‐19 pandemic.

*I worked with people who exercised from home during the covid pandemic and it helped me because I could exercise for my mental health too* [*P1*, *FG1*].

Participants recognised that spending time on an outdoor walk had benefits for their mental health and had subsequently advised other members of their community to engage in outdoor walks during the COVID‐19 pandemic, when their physical activity levels were at their lowest.

*I do feel like it helps in a lot of ways* [*…*] *it really does help sort of mental health and just put you in a little bit more of a positive kind of mood* [*P4*, *FG2*].

## 4. Discussion

This study explored barriers and facilitators to the self‐management of type 2 diabetes mellitus (T2DM) within the UK Chinese community. Key areas of exploration included dietary practices and physical activity engagement. Family, cultural values and social roles featured prominently, influencing both health behaviours and decision‐making. Barriers to healthy eating included time constraints and convenience, culturally embedded dietary practices, such as viewing food as a symbol of prosperity and lack of nutritional knowledge. Facilitators for healthy eating included family and social support, health education and dietary modifications. For physical activity, barriers included competing work and family commitments, a perceived lack of significance of exercise and environmental constraints, whereas facilitators included engaging in culturally familiar activities such as Tai Chi, engaging in community‐based exercise and exercising for benefits to mental health.

Social networks emerged as a central facilitator of diabetes management. This finding has been demonstrated in other chronic conditions, where social networks play an important role in supporting the management of these conditions [38, 39]. Furthermore, as in prior research with Chinese individuals in North America [40, 41], family relationships in this UK‐based sample played a significant role in shaping dietary and exercise behaviours. Zhang et al. [42, 43] similarly found that Chinese patients in the UK who perceived greater social support demonstrated better diabetes management.

The influence of collectivist values was evident, as participants often made health decisions in relation to family wellbeing. This aligns with research showing that collectivist cultures place the wellbeing of the family above individual needs [44]. While communal eating and celebration reinforced social cohesion, they also created tension when dietary restraint conflicted with expectations of hospitality and abundance. This tension reflects an important contradiction between collectivist norms and individual responsibility emphasised in UK diabetes self‐management advice [45]. Culturally sensitive interventions should therefore not position dietary change as an individual endeavour alone but incorporate family‐based education models that align with shared decision‐making practices.

Interestingly, stigma was not a dominant theme in our study. Participants were generally open about their diagnosis and did not report feelings of shame when disclosing their condition. In some cases, disclosure of their diagnosis was viewed as a protective strategy for maintaining healthier eating habits, reducing social pressure. This contrasts with prior research conducted among Chinese Americans, where stigma around diabetes, rooted in perceptions of poor self‐control, was more prominent [46, 47]. The relatively long illness duration among participants (many had lived with diabetes for over 3 years) may have contributed to normalisation over time. However, future research is needed to explore stigma dynamics within more recently diagnosed or younger Chinese populations.

It should be noted that translating international findings to the UK requires a critical lens. While international research offers foundational insights, the UK context introduces unique challenges such as language barriers, access to culturally appropriate healthcare and the negotiation of dual cultural identities [48]. These contextual dynamics may shape perceptions of illness, healthcare engagement and the feasibility of lifestyle changes.

The Health Belief Model (HBM) [43] provides a useful framework to interpret our findings. According to the model, health behaviour is shaped by perceived susceptibility, perceived severity, perceived benefits and perceived barriers. Participants with strong family histories of diabetes frequently described anticipating diabetes, reflecting heightened perceived susceptibility. Despite recognising susceptibility, some participants framed diabetes as a manageable condition, suggesting lower perceived severity. Many participants acknowledged that dietary modification could improve glycaemic control, indicating high perceived benefit, particularly for food‐related behaviours. Work demands, time constraints, limited culturally tailored advice and environmental factors were the most consistently cited perceived barriers. It is important to note that perceived barriers appeared to outweigh perceived benefits in relation to physical activity more than dietary change. Exercise was often viewed as less important than diet in relation to diabetes management. This may provide insight into differing views regarding the importance of physical activity.

The implications of this research extend to how diabetes services are structured and delivered in the UK. Participants consistently reported a lack of culturally tailored dietary guidance and uncertainty regarding healthy appropriate food replacements. Health professionals were often perceived as unfamiliar with Chinese cuisine, reducing the relevance and impact of dietary advice. Concrete strategies are therefore required. Bilingual (English–Mandarin/Cantonese) visual dietary guides should be developed to illustrate common Chinese staple foods (e.g., rice, dim sum, festival foods) with portion guidance and glycaemic impact indicators. Due to the importance of family and support networks, the incorporation of family‐inclusive diabetes education sessions may be helpful, as well as encouraging peer‐based support within community groups. This would help families where meals are prepared and eaten together. A study carried out in China provides support for this strategy. A study carried out by Zhong et al. [49] explored the role of peer support interventions where people with diabetes supported each other within primary care and community contexts. The study found that peer support improved lifestyle behaviours such as diet and exercise, resulted in better glycaemic outcomes compared to standard care alone and increased engagement with local services.

Furthermore, due to the preference of culturally appropriate exercise, partnerships with local Chinese community centres would be beneficial to combine group‐based education sessions with culturally familiar activities such as Tai Chi or healthy cooking demonstrations. This community engagement would support both the community and healthcare professionals. Participants also expressed motivation when exercise was socially embedded. Group‐based walking clubs, culturally familiar exercise sessions (e.g., Tai Chi) and diabetes peer support groups held within Chinese community venues may therefore represent feasible and culturally relevant intervention models. Additionally, the findings highlight the need for healthcare providers to receive cultural competency training and for materials to be co‐developed with the Chinese community to ensure resonance.

This study is, to our knowledge, the first to explore the barriers and facilitators of type 2 diabetes management among Chinese adults in the UK. Although the study provides some important insights into diabetes management in this community, there are some factors that should be highlighted for future research. Participants were recruited from Cambridge and Liverpool; therefore, future research should explore patterns across a larger geographical range due to the difference of community structures and healthcare access across the UK.

All focus groups were conducted via Zoom. Although videoconferencing has been seen as a convenient, cost‐effective and flexible and effective method of data collection [25]. Digital participation may have excluded individuals with limited digital literacy or access. Future research incorporating in‐person data collection through community organisations may enhance inclusivity.

Additionally, the sample was predominantly female (16 of the 22 participants) and over 50 years of age. This gender and age split within the sample may limit insight into the diabetes management of male participants or younger Chinese people with diabetes. Future research with participants from younger age groups and greater male representation would be beneficial. Additionally, member checking was not undertaken due to the logistical complexity of contacting participants across multiple language groups and locations. However, credibility was supported through sustained reflexive practice throughout the research process. The insider‐outsider dynamic within the research team facilitated both cultural sensitivity and critical distance in the analysis. Future research may consider incorporating participant feedback workshops or community dissemination events to further enhance collaborative trustworthiness.

## 5. Conclusion

This study provides novel insight into the cultural, familial and structural factors shaping type 2 diabetes self‐management among Chinese adults in the UK. While participants demonstrated awareness of dietary benefits and strong family support networks, perceived barriers, particularly in relation to physical activity and culturally relevant healthcare advice, remained substantial. The findings highlight the need for culturally responsive, family‐inclusive and community‐embedded diabetes services that move beyond individualised behaviour change models. By aligning healthcare delivery with collectivist values and lived experiences, UK diabetes services may enhance engagement, sustainability of behaviour change and ultimately health outcomes within this underserved population.

## Author Contributions

Conceptualisation, methodology: Tarnjit Sehmbi, Shahina Pardhan and Raju Sapkota. Data collection: Ran Cao, Tarnjit Sehmbi and Raju Sapkota. Data analysis: Tarnjit Sehmbi, Shahina Pardhan and Ran Cao. Writing – original draft: Tarnjit Sehmbi, Ran Cao, Shahina Pardhan and Raju Sapkota. Writing – review and editing: Tarnjit Sehmbi and Shahina Pardhan. Supervision: Shahina Pardhan.

## Funding

This research received no specific grant from any funding agency in the public, commercial or not‐for‐profit sectors.

## Conflicts of Interest

The authors declare no conflicts of interest.

## Data Availability

The data are available upon request.
